# Developing a learning tool for advanced life support and resuscitation: Performance Reflection Model for Resuscitation (PRM-Resus)

**DOI:** 10.1186/s12909-025-07509-9

**Published:** 2025-07-04

**Authors:** Yoriko Kikkawa, Leah McIntosh, Timothy J. Mavin, Melanie Barlow, Liam O’Brien, Steven Hodge, Sarah Janssens

**Affiliations:** 1 Griffith Institute for Educational Research & School of Education and Professional Studies, Griffith University, 176 Messines Ridge Road, Mt Gravatt, QLD, 4122 Australia; 2 Mater Education, Raymond Terrace, South Brisbane, QLD, 4101 Australia; 3 Institute for Learning Sciences & Teacher Education, Australian Catholic University, 1100 Nudgee Road, Banyo, QLD, 4014 Australia; 4 Faculty of Health Science, Australian Catholic University, 1100 Nudgee Road, Banyo, QLD 4014 Australia; 5 Queensland Alliance of Environmental Health Science, University of Queensland, 20 Cornwall Street Woolloongabba, QLD, 4102 Australia; 6 Mater Mothers’ Hospital Brisbane, Raymond Terrace, South Brisbane, QLD, 4101 Australia

**Keywords:** Interdisciplinary, Advanced life support, Simulation, Performance assessment, Non-technical skills, Team work

## Abstract

**Background:**

Acquiring proficiency in advanced life support (ALS) can pose challenges for novice learners. Simulation-based training (SBT) is widely used to address this, offering learners opportunities to practise and receive feedback during debriefing. However, existing performance tools often lack the clarity, behavioural specificity, and educational scaffolding required to support deep reflective learning. This study aimed to develop and evaluate the Performance Reflection Model for Resuscitation (PRM-Resus) and to integrate it with the ALS Team Model and structured video exemplars as a comprehensive learning package to enhance ALS training.

**Methods:**

The study involved four phases. Phase 1 created the ALS Team Model to clarify individual roles. Phase 2 focused on co-designing PRM-Resus, using team expertise and the Team Model to create behaviourally anchored performance descriptors. In Phase 3, video scenarios were produced to represent ALS team performance at varying proficiency levels. Phase 4 evaluated the PRM-Resus through expert think-aloud studies. Qualitative content analysis was used alongside Cronbach’s alpha to assess internal consistency and its use for SBT.

**Results:**

The PRM-Resus comprises four domains—clinical skills, clinical knowledge, team management, and leadership—each defined by behavioural descriptors across three performance levels. The participating experts endorsed the tool’s clarity, structure, and educational value for novice learners. Internal consistency was high (α > 0.95). When used alongside the ALS Team Model and video exemplars, PRM-Resus facilitated deeper performance analysis, which had potential for enhancing post-simulation reflection and supporting faculty development.

**Conclusions:**

This study presents a novel, interdisciplinary framework that integrates PRM-Resus, the ALS Team Model, and video exemplars to support reflective learning in ALS simulation. Together, these tools help novice learners build a concrete understanding of effective team performance and enable educators to deliver more structured feedback. Further research should explore its impact on learner development and potential translation into improved clinical outcomes.

**Supplementary Information:**

The online version contains supplementary material available at 10.1186/s12909-025-07509-9.

## Introduction

Effective management of medical emergencies requires healthcare professionals to develop competence across interrelated technical skills (e.g., procedural skills) and non-technical skills (NTS; e.g., behavioural skills such as communication and decision-making^1^). Healthcare programs increasingly include NTS training alongside technical skills, particularly for high-stakes interprofessional teams involved in advanced life support (ALS). While classroom-based activities are commonly employed to teach theoretical knowledge and clinical algorithms, simulation-based training (SBT) is universally recognised as an essential instructional method. SBT offers the opportunity to apply knowledge and skills in a contextualised environment [1, 2]. Furthermore, when SBT includes formative and summative performance assessments, feedback and guided reflection, the learning experience is significantly enhanced [3, 4].

To support learning, many tools have been developed to assess the technical and NTS of individuals and teams engaged in healthcare emergency responses [2, 5–9]. Accurate assessment is essential for providing learners with meaningful feedback that promotes reflection and learning., As Salas and his colleagues noted: “If performance is not explicitly defined and measured, it cannot be changed or improved in a systematic way. This is especially true in SBT, where many goals, purposes, and reasons drive the need to measure performance”. The literature broadly supports using tools for assessment and/or self-reflection to enhance learning, with evidence suggesting that such tools accelerate skill acquisition by helping learners identify and model desirable and undesirable behaviours, ultimately improving performance during resuscitation [3, 8].

A range of tools exist for assessing individual and team performance during both resuscitation training and real clinical scenarios. However, each tool presents its own benefits and drawbacks [5, 6, 8, 10]. Several criticisms have emerged in the literature, highlighting key challenges.

First, many tools struggle with the integrated assessment of technical skills, NTS, and team functioning [5, 11]. Despite the interdependent nature of these skill sets, most tools assess either technical skills or NTS performance. NTS-focused tools typically rely on guidelines to infer technical skill markers, whereas those attempting to assess both domains often become overly complex and cumbersome to use [5, 10]. For example, Peltonen and colleagues developed a comprehensive tool comprising 69 performance elements [5]. Yet, they acknowledged that its real time application was impractical given the inherent complexity of managing medical emergencies. Striving to reflect this complexity within a single tool can result in instruments that are too detailed for real-time use. Thus, a balance must be struck between providing sufficient detail to support critical performance analysis and ensuring usability for learners and instructors.

Second, the interconnectedness between individual and team performance is often overlooked. Many tools assess either the performance of individuals within a team (e.g., the team leader) or the team as a whole [6]. Tools focused solely on individuals may ignore how each person’s actions influence team outcomes, while team-focused tools may fail to account for individual contributions. It is essential to assess both levels; however, current tools rarely capture the bidirectional influence between individual and team performance. A tool that enables seamless movement between these two levels of analysis is needed.

Third, existing tools utilise various scoring strategies, including dichotomous assessment—Likert-like scales and behaviourally anchored rating scales (BARS). While BARS can be wordy and complex, they offer observable behavioural markers and standardised vocabulary for interpretation of performance [8]. Simpler assessments, such as dichotomous yes–no tools, though easier to use, often lack the depth to guide improvement strategies [6]. No single scoring strategy is inherently superior; tools must balance simplicity with the ability to differentiate suboptimal, acceptable and aspirational performance.

Although these are not the only challenges facing performance assessment in resuscitation, they were central to the authors’ considerations during a three-year research program aimed at improving ALS training for healthcare workers during the COVID-19 pandemic. Specifically, the authors sought to design a video-augmented training program to support skill acquisition and application among novice ALS learners. Developing a fit-for-purpose assessment tool to support learner reflection and analysis became a cornerstone of the study.

Leveraging the interdisciplinary and cross-sector expertise across healthcare, higher education, aviation (both civil and military) [4, 12], the team developed the Performance Reflection Model for Resuscitation (PRM-Resus) alongside tailored ALS training materials. The model’s development was informed by principles from aviation studies by Mavin and his colleagues, including: (a) integrating technical and NTS performance rather than assessing them separately and ideally avoiding the labels technical and NTS altogether [13]; (b) avoiding separate assessment of teamwork, viewing it instead as an emergent outcome of individual skills [14]; (c) identifying individual skills critical to effective ALS performance [15]; (d) using “word pictures” to describe varying levels of performance—average, good, and excellent performance [15]; and (e) evaluating the tool prior to broad-scale implementation [15, 16].

This paper now describes the development and evaluation of the Performance Reflection Model for Resuscitation (PRM-Resus), designed to address some limitations of existing tools. The ultimate aim was to create an integrated, reflective model that enhances SBT by supporting ALS novice learners and instructors in analysing the interplay between technical and NTS, thereby improving instructional delivery and learner development in ALS training programs.

## Method

The study site was a large private hospital in Queensland, Australia, which provided access to its nationally accredited, hospital-based simulation facility. The simulation facility had separate classrooms and two fully interactive hospital room simulation suites. The simulated environment was operated by staff behind a one-way mirror, with integrated audio-visual recording equipment. The participants were healthcare staff employed at the site. All participants provided informed consent prior to participation, in accordance with institutional ethical review guidelines (see more details in the Declaration section).

**Fig. 1:**
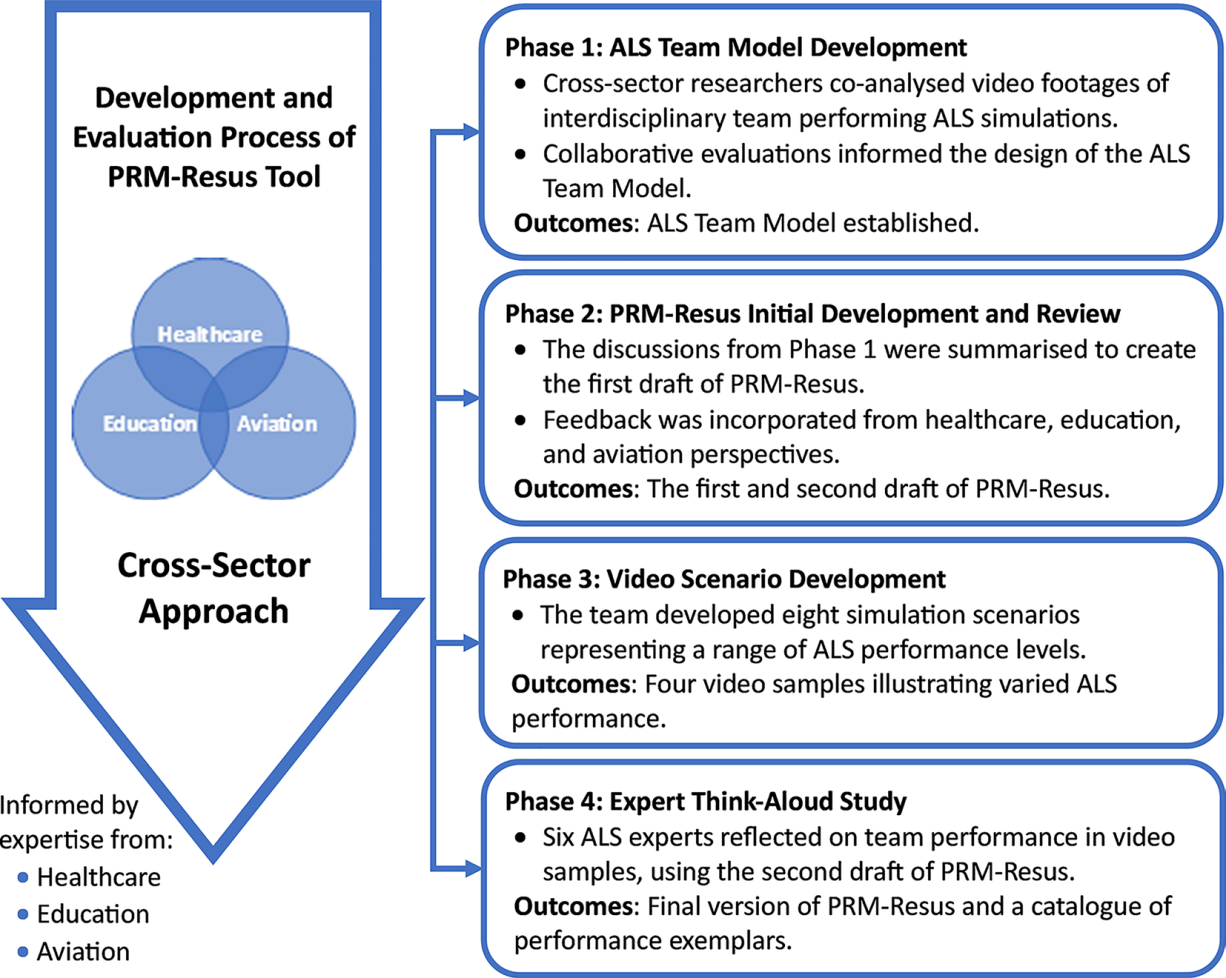
The overall process of cross-sector development and evaluation of the PRM-Resus comprises four iterative phases. The tool was designed to support ALS novice learners by guiding reflection and performance analysis. It is intended for use alongside the ALS Team Model, video scenarios as training videos, and a catalogue of performance exemplars

A mixed-method approach [17] was applied to four distinct but interrelated phases to develop and evaluate the PRM-Resus for SBT (Fig. 1). The phases included: ALS team model development; PRM-Resus development and review; video scenario development; and expert think-aloud video reviews. The research team, with various backgrounds in healthcare, education, and aviation, engaged continuously in all phases to investigate elements of ALS resuscitation team performance from cross-sector perspectives.

### Phase 1: ALS team model development

In response to the COVID-19 pandemic, the site hospital developed and implemented new ALS procedures for infectious patients. To promote staff integration of COVID-19 infection control protocols into the existing ALS procedures, a full-day SBT course was developed. The course covered classroom-based instruction and four separate simulation scenarios, followed by post-simulation debriefings. The simulation scenarios included two standard (non-infectious) and two pandemic (COVID-19 specific) cases. A total of three courses were completed, and all simulation and debriefing sessions were video recorded. From twelve course scenario videos, four were selected by the research team to represent a range of performance levels. These were referred to as *Video Set A* and used to develop the ALS Team Model.

Four research members met online via Microsoft Teams to collaboratively review and analyse performance in Video Set A, contributing to the co-design of the model. This method was similar to the approaches used in applied education and simulation-based research that emphasise collaborative video analysis to facilitate shared interpretation and co-construction of meaning among interdisciplinary teams. Specifically, it reflects methods used to promote authentic engagement and model development through simulation in health-related fields [e.g., 18].

During these sessions, the educational researcher (YK) facilitated the discussion by asking healthcare researchers (LM, MB, LO)—all experienced ALS instructors—to rate team performance (poor, good, excellent) and explain the reasoning behind their judgements. These collaborative analysis sessions were recorded and transcribed to capture the team’s evaluative dialogue accurately. The transcriptions were then synthesised to inform the initial development of the ALS Team Model.

Although this approach does not constitute a formal focus group or Delphi study, it aligns with established practices in interdisciplinary educational research. Similar collaborative analysis techniques have been employed in teacher education and healthcare training to support reflection, shared understanding, and tool development [19–21]. Importantly, this Phase was an ongoing process throughout the study as the later activities informed the improvements of the ALS Team Model.

### Phase 2: PRM-Resus initial development and review

Through summarising the discussions of team analysis in Phase 1, key elements (e.g., individual skill sets, communication, task management) were extracted for a range of ALS performance (poor, good, excellent), leading to the development of the first draft of PRM-Resus. This draft was emailed to the research team (*n* = 6), who provided feedback based on their healthcare, education, and aviation expertise. For example, LM (nurse and ALS instructor) reviewed ALS guidelines and resuscitation-related literature to add more clinical components, SJ (Director Obstetrics and Gynaecology and ex-director of the simulation centre) contributed insights to leadership and team management, and TM (pilot and educator) incorporated aviation-based knowledge of performance assessment. This feedback informed the second draft of the PRM-Resus.

### Phase 3: Video scenario development and refinement of PRM-Resus

**Fig. 2:**
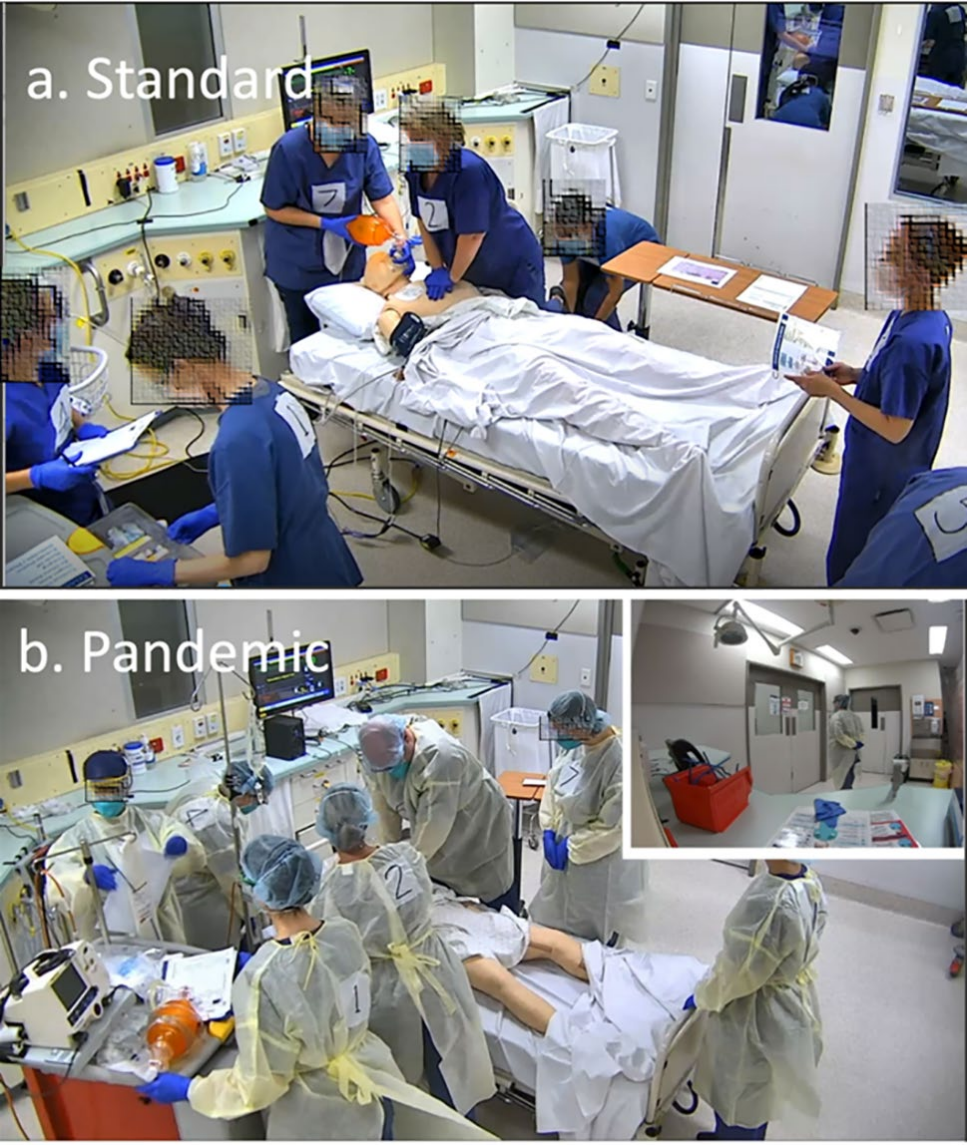
Video Set 2 shows examples of both standard and pandemic ALS scenarios. Note: Pandemic ALS also provided footage from outside the room

The research team translated the second draft PRM-Resus into eight video samples of ALS performance (4 x standard, 4 x pandemic) that were filmed with seven ALS experts. During filming, each expert wore appropriate PPE and was numbered for ease of identification (see Fig. 2). The filming approach mirrored aviation video development, in which scenarios were performed without rehearsal, reflected upon in a debrief, and then performed again. [15], allowing natural improvement in performance while maintaining realism. This process resulted in a series of videos with progressively improved, yet authentic performance. Adobe Premiere Pro was used to synchronise footages from four camera angles to produce the most representative version of each scenario. Four final videos (2 x standard, 2 x pandemic), referred to as *Video Set B*, were created from this series. All debriefing conversations were recorded and transcribed to provide further refinements of the PRM-Resus.

### Phase 4: Expert think-aloud study: final refinement of PRM-Resus and example extractions

The research team employed a paired think-aloud method to examine how ALS experts assessed the performance of Video Set B. This approach, commonly used in aviation and cognitive science research, allows experts to articulate their thought processes during performance evaluation [15]. While not traditionally used in healthcare, it was adapted to support cross-disciplinary insights into ALS performance assessment. For this study, six pairs of ALS experts (three nurse pairs, two doctor pairs, and one mixed nurse–doctor pair) were asked to assess the performance in each video. Participants were purposefully selected for their senior clinical roles and a minimum of 12 months of experience as ALS instructors, ensuring they could meaningfully contribute to the co-design and evaluation of the PRM-Resus.

Each pair was provided with a single copy of the PRM-Resus and asked to assess each video, while discussing their rationale jointly. The room was arranged so cameras could record areas highlighted on the PRM-Resus, the specific video segment being discussed, and the accompanying conversation (see Appendix A).

Content analysis was employed to identify key moments emphasised by each pair during video review. Particular attention was paid to the video segments where the pairs discussed multiple performance categories simultaneously. This focus reflected a core principle of the study: while individual competencies—such as technical skills and NTS—can be assessed separately, their true value lies in how they interact to support overall team performance [16]. The list of performance examples was created for future training as part of the host project. The examples consisted of the selected segment of video training and the comments from the experts’ conversation from their think-aloud sessions.

In addition to the qualitative analysis, Cronbach’s alpha coefficient, a frequently used measure when evaluating and refining new scales, was calculated to evaluate the internal consistency of the PRM-Resus [22]. Cronbach’s alpha was calculated across all conditions (good and poor performance in both the standard and pandemic scenarios) to assess how well the items measured the constructs under study and to strengthen the overall rigour of the evaluation process. The analysis used SPSS version 23. Each of the three subscales was evaluated: technical skills (seven items), team management (five items), leadership (four items).

## Results

### Phase 1: ALS team model development

**Fig. 3:**
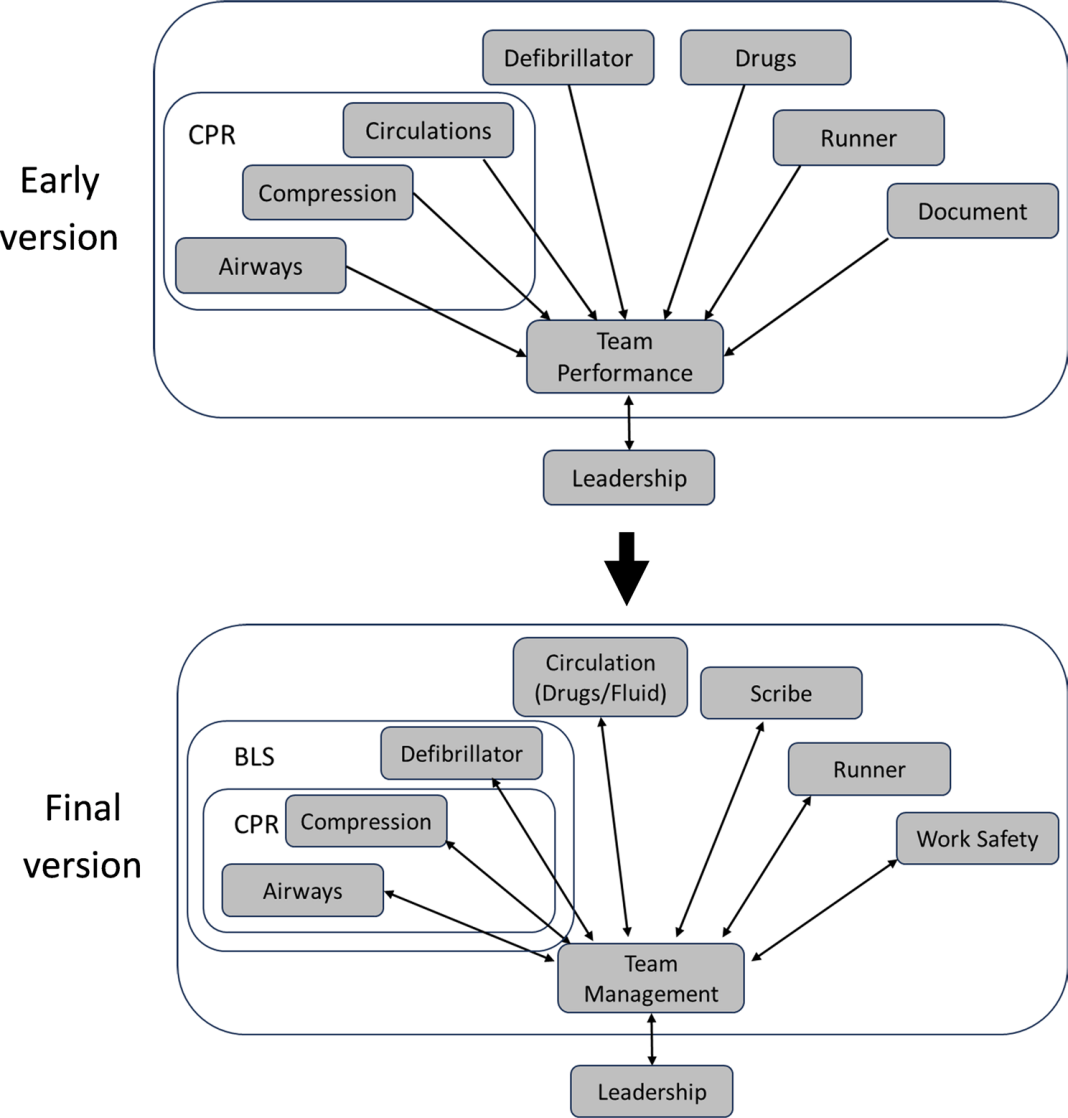
Early and final versions of the ALS Team Model. It included seven clinical roles—airway, compression, defibrillator, circulation (drugs and fluid), scribe, runner, work safety—to be managed as a team and one leadership role to manage the team

Initial analysis of Video Set A data revealed that the healthcare researchers encountered difficulty determining team performance due to the high number of variables. Leveraging off aviation research [23] and the team’s recent cross-sector analysis of video footages in maternity emergency management [21], the team shifted focus to individual roles only. Presenting a pictorial representation of the roles—referred to as the ‘ALS Team Model’ (Fig. 3)—facilitated mutual understanding of ALS across the clinical, education and aviation team members. The ALS Team Model evolved from Phase 1, with continual modifications occurring as the project progressed through later phases. The following provides an example of how the ALS Team Model evolved.

#### Example 1: Refining cardiopulmonary resuscitation (CPR) and basic life support (BLS)

During the early stages of Phase 1, the healthcare team often discussed CPR. However, the aviation/education team asked, “What actually is CPR?”. This initiated a discussion highlighting the tight link between the ‘airway’ and ‘compression’ roles, conceptualised as a micro-team within the ‘CPR’ subset of roles. Further, CPR was closely tied to the defibrillation role, forming a Basic Life Support (BLS) subset of roles. The Team Model was adapted to reflect these subsets of CPR and BLS (Fig. 3). Further refinements occurred in Phase 4 based on the participating experts’ reflections on the ALS Video Set B (see Quote 1 in Appendix B).

#### Example 2: Integrating the circulation and drugs roles

Initial versions of the model had circulation and drugs as separate roles. However, analysis by ALS team members revealed that typical ALS scenarios do not require a dedicated ‘fluid’ role—except in specific cases (e.g., hypovolaemia or active bleeding), unlike other disciplines that might require a consistent fluid supply to the patient, like maternity cases. These roles were therefore combined in the final version of the ALS Team Model.

#### Example 3: Expanding the role of work safety

The original version assigned work safety only to pandemic scenarios, based on team consensus that it was a dedicated role focused on staff safety measures related to PPE—application, breach monitoring, and removal. However, expert conversations in Phase 4 affirmed that work safety is also relevant to standard scenarios, recognising that all team members share responsibility for work safety. Under certain circumstances (e.g., additional precautions), it may be designated as a stand-alone role (see Quote 2 in Appendix B). Consequently, work safety was added to both standard and pandemic scenarios.

#### Example 4: Refining team management, leadership, and whole team interaction

During Phase 1, healthcare researchers emphasised the importance of effective workforce utilisation, specifically assigning roles based on individual experience and expertise. This viewpoint highlighted the influence of team management on role performance (see Quote 3 in Appendix B). By further delineating aspects of team management, the team could better identify how one role’s performance influenced others—such as switching compressions due to fatigue or verifying drug administration. Similarly, team management and leadership (e.g., global awareness, team structure, communication) were viewed as interrelated and synergistic. To reflect this, arrows connecting areas in the model were made bidirectional, representing mutual influence between individual roles and team leadership.

### Phase 2: PRM-Resus initial development and review

The ALS Team Model was used in parallel to inform the development of the PRM-Resus. Performance was described across four categories: clinical skills, clinical knowledge, team management and leadership, each with associated performance elements. Clinical skill elements included the seven roles of airway, compression, defibrillator, circulation, scribe, runner, and work safety. Clinical knowledge remained the sole category that informed each of the clinical roles. Team management performance elements included role allocation/workload, self-control/influence, handover, communication/team membership, and team cohesion/atmosphere/cooperation. Leadership performance elements included global awareness/workload or control of others, team structure, problem-solving/escalation/decision-making, and communication.

**Fig. 4:**
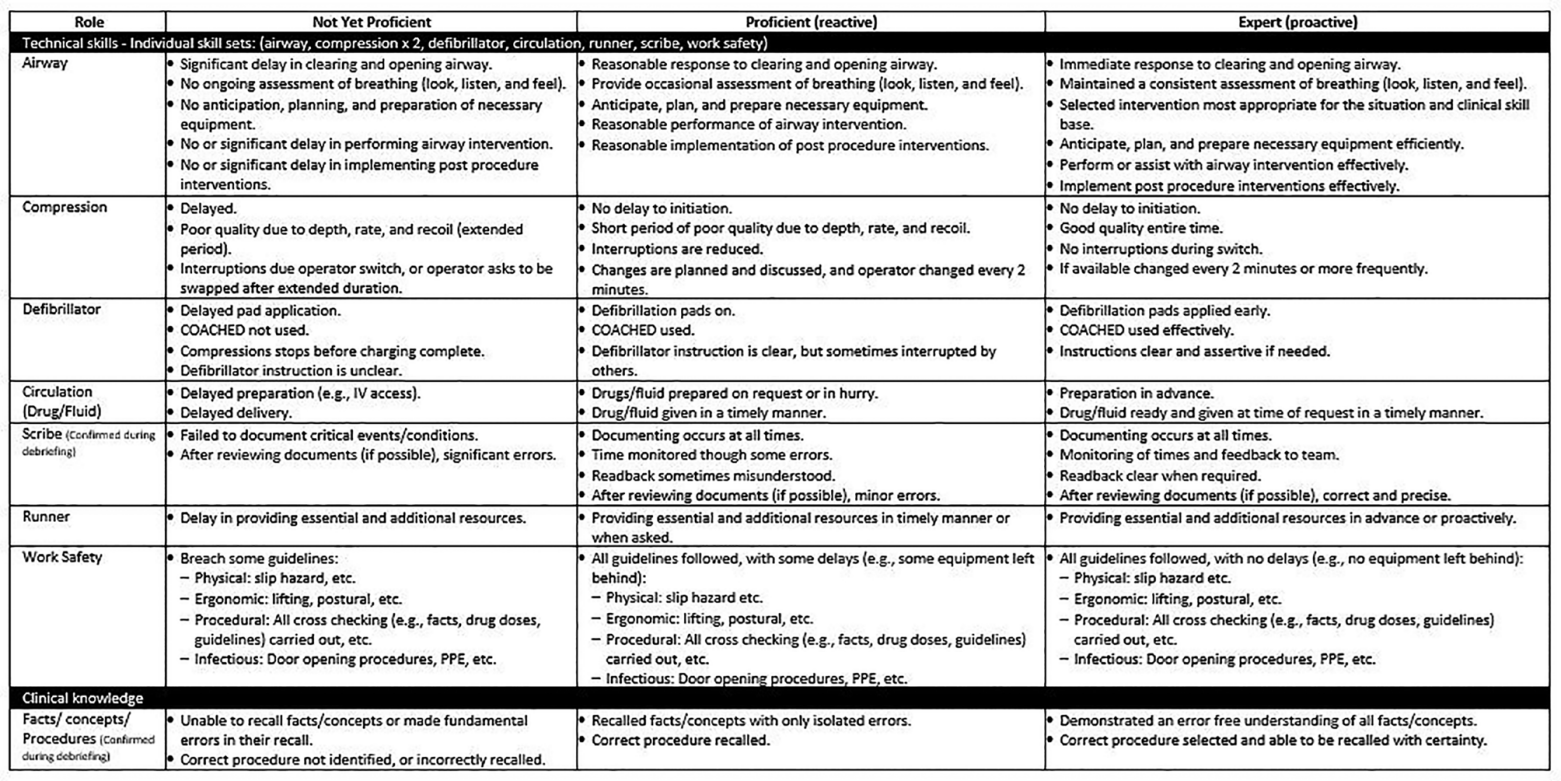
Page one of the final version of the PRM-Resus

**Fig. 5:**
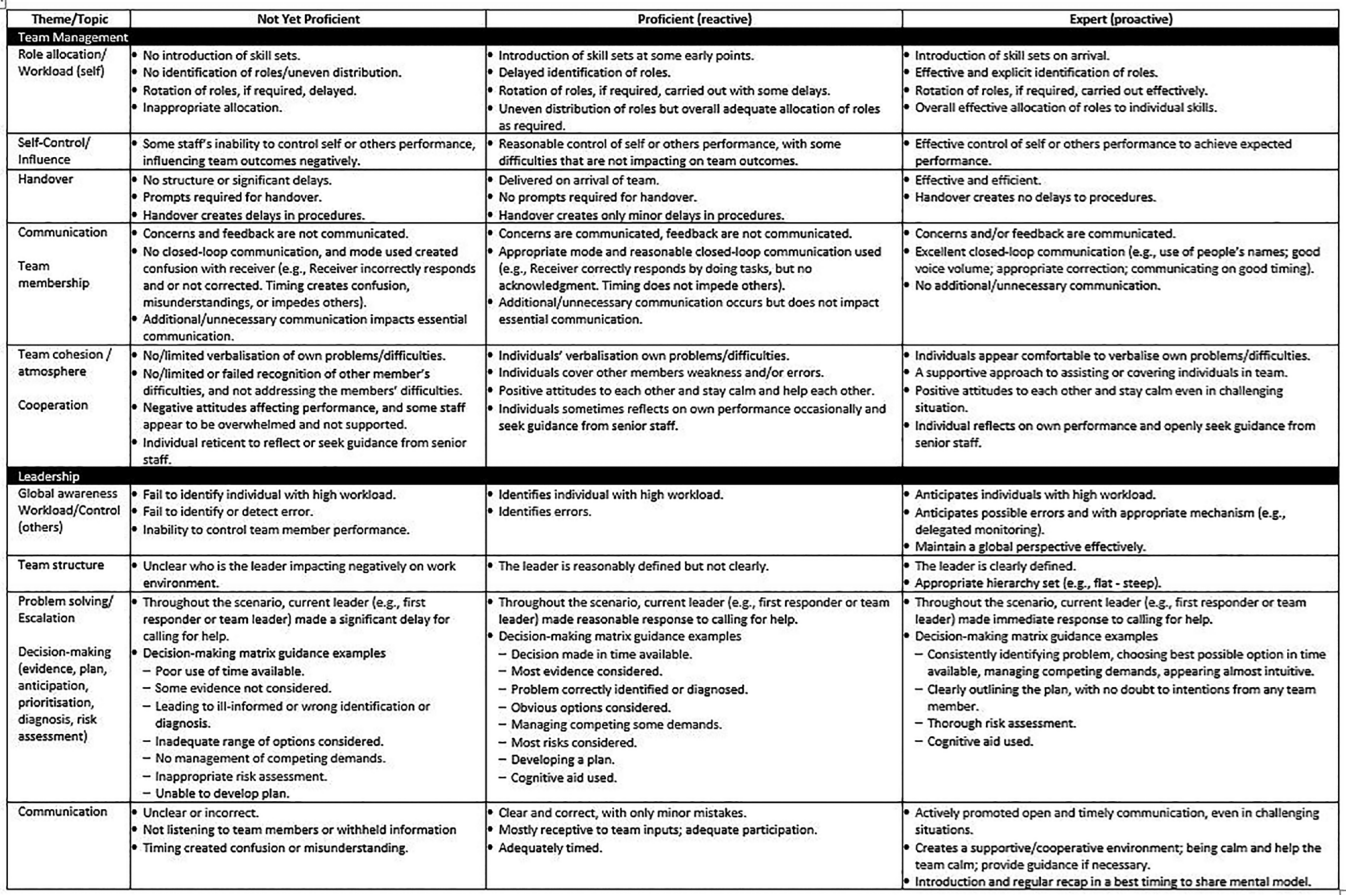
Page two of the final version of the PRM-Resus

For each performance element (e.g., airway), “word pictures” were developed to describe three levels: (1) Not Yet Proficient, (2) Proficient, and (3) Expert Performance. Expert interviewees received the three-level scale well, especially for educational use. Figures 4 and 5 show the final version of the PRM-Resus.

### Phase 3: Video development

In Phase 3, the conversations of post-film debriefings highlighted the focus of each filming attempt. These comments provided refinement of PRM-Resus and guided the selection of four videos from the eight produced. For both standard and pandemic scenarios, the first attempt was chosen to present not-yet-proficient performance, and the third attempt was for expert performance.

### Phase 4: Expert think-aloud study

A total of 12 ALS experts participated in the think-aloud study (see Table 1). After briefly explaining the study, each pair assessed four separate videos. The PRM-Resus scores were recorded (Table 2). Content analysis was then conducted to derive key moments (Table 3). These key moments were used to: (a) evaluate the PRM-Resus effectiveness, (b) inform modifications to the ALS Team Model and the PRM-Resus, and (c) explore the tool’s potential use in training.

**Table 1:** Background information of the participants

Pair	Qualification	Current profession	Stream/Hospital/area	Total clinical experience
Pair 1	Nurse	Healthcare educator	Critical care	10–14 years
	Doctor	Healthcare educator	Critical care	15 years +
Pair 2	Nurse	Healthcare educator	Critical care	15 years +
	Nurse	Healthcare educator	Critical care	15 years +
Pair 3	Nurse	Healthcare educator	Critical care	4–9 years
	Nurse	Nurse	Critical care	10–14 years
Pair 4	Nurse	Clinical facilitator	Critical care	15 years +
	Nurse	Healthcare educator	Medical and surgical	4–9 years
Pair 5	Doctor	Medical officer	Maternity	15 years +
	Doctor	Medical officer	Critical care	15 years +
Pair 6	Doctor	Medical officer	Critical care	10–14 years
	Doctor	Medical officer	Critical care	10–14 years

**Table 2:** Overall PRM results

Scenario	Pair	Technical Skills							Clinical Knowledge	Team management					Leadership				Total
		Airway	Compression	Circulation (Drug/Fluid)	Defibrillator	Scribe	Runner	Work Safety		Role Allocation/ Workload(self)	Self-Control/Influence	Handover	Communication/Team Membership	Team Cohesion/Atmosphere/Cooperation	Global Awareness/Workload/Control(others)	Team Structure	Problem Solving/Escalation/Decision-making	Communication	
Standard (Good)	Pair1 (N/D)	3	3	3	3	3	3	3	3	3	3	3	3	3	3	3	3	3	
	Pair2 (N/N)	3	3	3	3	3	3	-	3	3	3	3	3	3	3	3	3	3	
	Pair3 (N/N)	3	3	3	3	2	3	3	2	3	3	3	3	3	3	3	3	3	
	Pair4 (N/N)	2	3	3	3	3	-	2	3	3	2	3	3	3	3	3	3	3	
	Pair5 (D/D)	3	3	3	3	3	3	3	3	3	3	3	3	3	3	3	3	3	
	Pair6 (D/D)	2	2	3	3	2	2	3	2	3	3	2	2	2	3	3	2	3	
	*Mean*	*2.67*	*2.83*	*3.00*	*3.00*	*2.67*	*2.80*	*2.80*	*2.67*	*3.00*	*2.83*	*2.83*	*2.83*	*2.83*	*3.00*	*3.00*	*2.83*	*3.00*	*2.86*
	*SD*	*0.52*	*0.41*	*0.00*	*0.00*	*0.52*	*0.45*	*0.45*	*0.52*	*0.00*	*0.41*	*0.41*	*0.41*	*0.41*	*0.00*	*0.00*	*0.41*	*0.00*	*0.29*
Pandemic (Good)	Pair1 (N/D)	3	3	3	3	2	3	3	2	3	3	3	3	3	3	3	3	3	
	Pair2 (N/N)	3	3	3	3	2	3	3	3	3	3	3	3	3	3	3	3	3	
	Pair3 (N/N)	3	3	3	3	2	3	3	3	3	3	3	3	3	3	3	3	3	
	Pair4 (N/N)	3	3	2	1	3	3	2	3	3	3	3	3	2	3	3	3	3	
	Pair5 (D/D)	3	3	3	3	3	3	3	3	3	3	3	3	3	3	3	3	3	
	Pair6 (D/D)	3	3	3	1	3	3	3	3	3	3	3	2	3	3	3	3	3	
	*Mean*	*3.00*	*3.00*	*2.83*	*2.33*	*2.50*	*3.00*	*2.83*	*2.83*	*3.00*	*3.00*	*3.00*	*2.83*	*2.83*	*3.00*	*3.00*	*3.00*	*3.00*	*2.88*
	*SD*	*0.00*	*0.00*	*0.41*	*1.03*	*0.55*	*0.00*	*0.41*	*0.41*	*0.00*	*0.00*	*0.00*	*0.41*	*0.41*	*0.00*	*0.00*	*0.00*	*0.00*	*0.21*
Standard (Poor)	Pair1 (N/D)	1	1	1	1	1	1	1	1	1	1	1	1	1	1	2	1	1	
	Pair2 (N/N)	2	1	2	2	2	1	-	2	1	1	1	2	2	1	1	1	1	
	Pair3 (N/N)	3	1	2	1	2	-	1	2	2	1	3	2	2	1	2	2	2	
	Pair4 (N/N)	2	1	1	1	2	-	1	1	1	1	1	1	1	1	2	1	1	
	Pair5 (D/D)	2	2	2	1	2	-	1	1	2	1	1	1	1	2	2	1	1	
	Pair6 (D/D)	2	1	2	1	2	2	1	2	1	1	2	1	1	1	2	1	1	
	*Mean*	*2.00*	*1.17*	*1.67*	*1.17*	*1.83*	*1.00*	*1.00*	*1.50*	*1.33*	*1.00*	*1.50*	*1.33*	*1.33*	*1.17*	*1.83*	*1.17*	*1.17*	*1.36*
	*SD*	*0.63*	*0.41*	*0.52*	*0.41*	*0.41*	*0.00*	*0.00*	*0.55*	*0.52*	*0.00*	*0.84*	*0.52*	*0.52*	*0.41*	*0.41*	*0.41*	*0.41*	*0.41*
Pandemic (Poor)	Pair1 (N/D)	1	1	1	1	1	1	1	1	1	1	1	1	1	1	1	1	1	
	Pair2 (N/N)	2	1	1	2	2	1	2	2	1	1	2	2	2	2	2	1	1	
	Pair3 (N/N)	1	1	1	1	1	2	1	1	1	2	2	2	1	1	2	1	1	
	Pair4 (N/N)	3	1	2	1	2	2	1	1	1	2	1	1	2	1	2	1	1	
	Pair5 (D/D)	1	1	1	1	1	3	1	1	1	1	2	1	1	1	2	1	1	
	Pair6 (D/D)	1	1	2	1	3	2	2	1	1	1	2	1	1	1	2	1	1	
	*Mean*	*1.50*	*1.00*	*1.33*	*1.17*	*1.67*	*1.83*	*1.33*	*1.17*	*1.00*	*1.33*	*1.67*	*1.33*	*1.33*	*1.17*	*1.83*	*1.00*	*1.00*	*1.33*
	*SD*	*0.84*	*0.00*	*0.52*	*0.41*	*0.82*	*0.75*	*0.52*	*0.41*	*0.00*	*0.52*	*0.52*	*0.52*	*0.52*	*0.41*	*0.41*	*0.00*	*0.00*	*0.42*

**Table 3:** Key moment examples of each theme from content analysis. These examples were also designed to provide the performance exemplars for learning activities in the ALS training program at the site hospital

Positive/ negative	Description	Case
**Clinical knowledge influencing role performance**		
-	Lack of defibrillator knowledge delayed the delivery of first shock	Standard Case 2
-	Lack of airway knowledge delayed the delivery of intubation	Standard Case 2
-	The drug person gave the incorrect dosage of amiodarone to the patient.	Standard Case 1
+	The team demonstrated the ALS algorithm effectively without errors.	Pandemic Case 1
**Interrelationships among individual roles and team management**		
-	Lack of defibrillator knowledge required other staff help the staff. It caused side communications among these staff, resulting in increase in noise level of the room. No one had attention to handover between the first responder staff and team leader.	Standard Case 2
+	Clear instructions from defibrillator role helped the team to prepare for rhythm analysis (+/- defibrillation).	Standard Case 1; Pandemic Case 1
+	Two staff worked collaboratively to complete the intubation of the airway. The task performance requires two people and communication and cooperation between them.	Standard Case 1; Pandemic Case 1
+	Airway and compression persons gave feedback about each other’s performance (i.e., quality of compression, chest rise)	Standard Case 1; Pandemic Case 1
-	The staff positioned her hand at a wrong spot for her compression, which was not corrected by airway person.	Standard Case 2
-	Defibrillator delivered a shock without clear visibility of the environment and people. This defibrillator’s unsafe performance impacts on work safety. At the same time, the team did not cooperate to give a clear view to Defibrillator, and team leader did not take control of the performance.	Standard Case 2
-	Runner did not communicate with the team or leader about what she was going to do before leaving the room. The team or leader did not realise they lost one team member.	Standard Case 2.
+	Team members checked PPE for each other to ensure work safety.	Pandemic Case 1 & 2
**Interrelationships between team management and leadership**		
+	Defibrillator/Scriber person raised her concern about the timing of drugs to team leader.	Standard Case 1
+	Airway person shared her plan of intubation to team leader.	Standard Case 1
-	Team members interrupted team leader’s recap/plans/instructions.	Standard Case 2; Pandemic Case 2
+	Team members stopped conversations and listen to team leader’s recap/plans/instructions.	Standard Case 1; Pandemic Case 1
+	Team leader confirmed individual roles immediately after entering the room and prioritised tasks (e.g., putting defib pads on, air bag on)	Standard Case 1
-	Team leader did not confirm individual roles, or team members did not introduce their skillsets at the beginning. It caused a chaotic situation where members switched roles multiple times, resulting in the delay in delivering the first shock.	Standard Case 2
+	Team leader identified individual roles in donning and doffing room before entering the room. Therefore, the team rapidly settled to establish the role distribution effectively immediately after entering the room.	Pandemic Case 1
+	Team leader confirmed two members to swap compression roles between them.	Standard Case 1
-	There was no communication regarding who and when to switch compression persons at the beginning. Lack of planning for rotation of compression operator, caused delays in transitioning.	Standard Case 2
+	Team leader used good volume voice and members’ names to give explicit instructions in the room.	Standard Case 1; Pandemic Case 1
+	Team leader verbalised her plan about when to have handover and prompted handover at the appropriate timing.	Standard Case 1; Pandemic Case 1
-	Team leader prompted handover at the same time as team members were trying to establish their roles and deliver the first shock delaying the delivery of the first shock and missing the delivery of drugs.	Standard Case 2
+	Team leader verbally acknowledged defibrillator’s prompt of starting rhythm analysis.	Standard Case 1; Pandemic Case 1
+	Team leader asked drug person to verbalise what drug was given to the patient for tracking and scribe documentation	Pandemic Case 1

Cronbach’s alpha coefficients for each subscale demonstrated high internal consistency of the tool across the conditions. The technical skills subscale consisted of 7 items (α = 0.99), the team management subscale consisted of five items (α = 0.96), with the leadership subscale composed of 4 items (α = 0.97). The Leadership subscale had the highest reliability, with the clinical subscale showing the lowest.

### Synthesis of findings

Overall, the Phase-4 expert interviews confirmed that the structure of the PRM-Resus—progressing from individual roles to clinical knowledge, team management, and leadership—was logical and intuitive. The expert participants emphasised that clinical knowledge could only be meaningfully assessed when demonstrated through observable actions. Consequently, high-performing videos were easier for the participants to rate because the participants’ behaviours closely aligned with the PRM-Resus descriptors. One participant noted, “it was straightforward” (StandardCase1_Nurse1), with minimal discussion required when behaviours matched the structured word pictures.

Phase 4 expert participants viewed the PRM-Resus as a valuable tool for post-simulation reflections, although they acknowledged that time constraints might limit its use during real-time debriefings. One of the tool’s key advantages that they identified was its potential to engage simulation observers in analysing performance more systematically. By shifting attention from vague overall team ratings (e.g., average or excellent) to specific role-level behaviours, the PRM-Resus helped make performance more observable, interpretable, and actionable. The Phase-4 participants also valued the tool’s capacity to analyse and articulate what constitutes effective ALS team performance. As one doctor remarked:

There’re a lot of buzz terms, like non-technical skills. A lot of people say, “We’ll understand it”, but then other people go, “That’s a great term—but what does that actually mean in reality?” [PRM-Resus] really articulates that out for them. I hate buzzwords if you can’t [explain what they mean or tell people how to recognise them]. (StandardCase2_Pair1)

Three key themes were identified through content analysis of expert think-aloud conversations: (1) clinical knowledge influences role performance, (2) interrelationships exist between individual roles and team management, and (3) interrelationships exist between team management and leadership. These themes were especially relevant in the context of SBT for ALS novice learners, where building an understanding of how technical skills and NTS, individual role and team management, and team management and leadership factors interact is critical. The following section further outlines how these interconnected performance elements contribute to the overall team performance.

### Theme 1: Clinical knowledge influences role performance

**Fig. 6:**
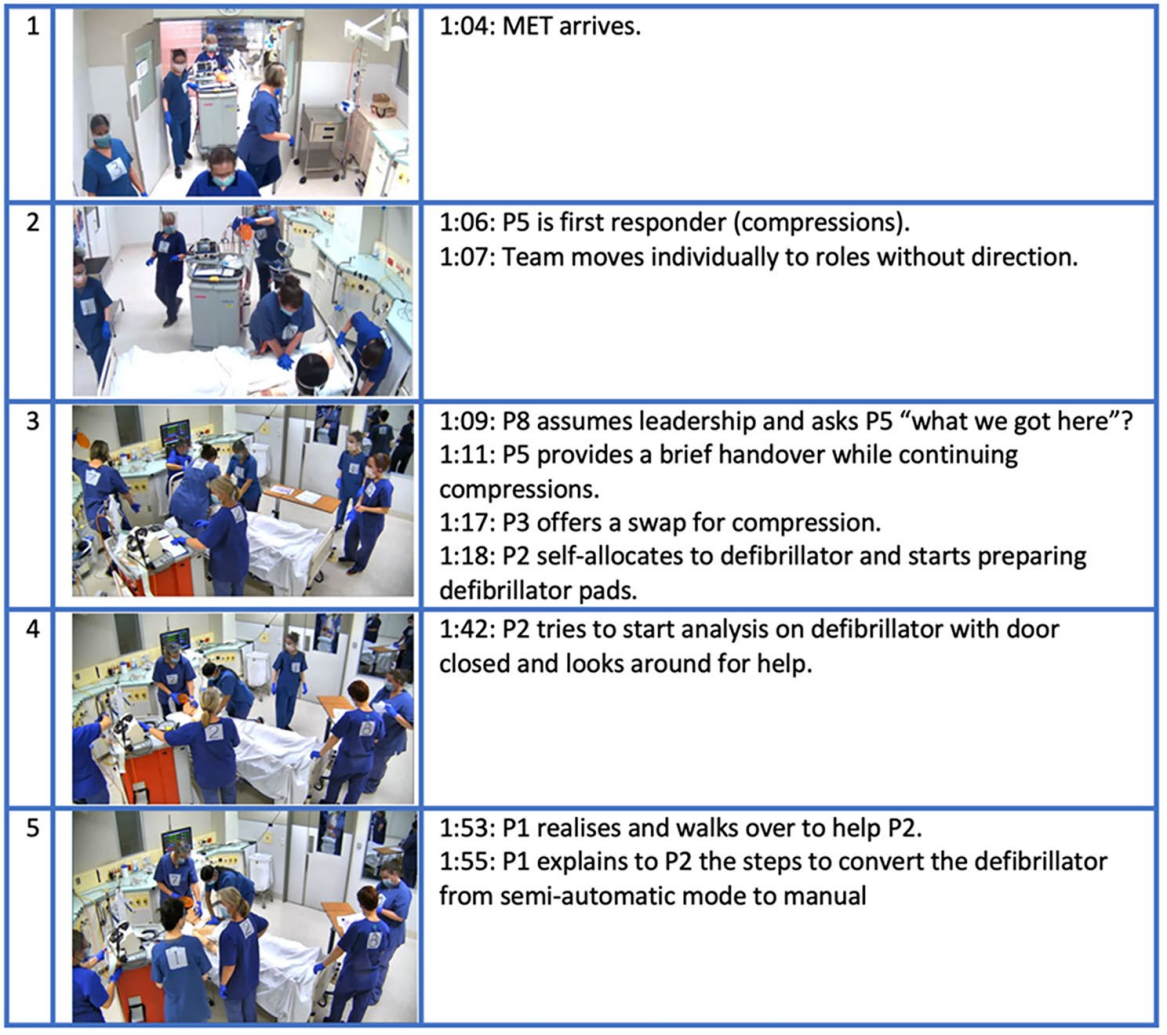
Clinical knowledge influencing role operational performance

PRM-Resus effectively illustrated how variations in clinical knowledge could significantly affect role performance. In one example (Standard Case 2), a staff member operating the defibrillator appeared unfamiliar with the equipment, resulting in a delayed rhythm analysis. This excerpt illustrates that a lack of clinical knowledge was the reason for average performance (see Fig. 6).

Nurse 3: The defib’s on, but I don’t see anyone doing anything with it. They’re just all talking amongst themselves, and there’s a significant delay in analysing.

Nurse 2: Well, the pads went on quick, and I’m thinking…

Nurse 3: I saw P2 (Defibrillator) was saying, “I don’t know what’s happening”. Someone else had to come in and open the door. But P4 and she went over to open the door.

(Standard Case 2_Pair 2)

### Theme 2: Interrelationships between role performance and team management

The Phase-4 participants highlighted the interplay between individual role performance and team management, especially in underperforming teams. In Standard Case 2, the team leader failed to identify that chest compressions were ineffective, which Expert Pair 1 described as “quite chaotic”. This situation was compounded by a nurse with limited ALS experience who delayed the delivery of the first shock, further disrupting flow.

Pair 1 observed that frequent switching between Defibrillator, Runner, and Airway roles, and the absence of formal skillset introductions compromised situational awareness and team coordination. The failure to establish a clear role allocation led to uneven task distribution—some staff were overloaded while others remained passive—contributing to critical delays.

Nurse 1: Let’s look at this (Team Management section). So, this is where I guess all the root cause issues are…. Where does role allocation happen? It happens at the 4-minute mark, isn’t it?

Doctor 1: Probably after the first shock…. There’s a delay to that defib, and they eventually [started allocating roles]. No one introduced their skillsets.

Nurse 1: No, and this is the point…Nobody introduces themselves to the room. There’s no clear establishment of any of their roles. Even on entry, [someone should’ve] said “I’m team leader”—but there’s none of that.

Doctor 1: The majority of these points [fall under] ‘not yet proficient’. There was a delayed identification of roles…. It didn’t meet any of these other categories here to suggest it was good. They kept switching due to P2’s inappropriate role.

Nurse 1: I totally agree. It’s not just necessarily no identification or uneven distribution. My concern is that there’re two people who are doing a lot, [whereas] this P3 spends most of the time just sitting there staring at. I didn’t actually see her do much at all other than jump in to do about 1.5 min of CPR. So that’s concerning for me.

(StandardCase2_Pair1)

### Theme 3: Interrelationships between team management and leadership

The Phase-4 participants identified several issues linked to leadership and team management, including the absence of a clear leader, poorly timed handovers, team inaction due to a lack of direction, insufficient clinical knowledge, and interruptions during rhythm analysis. They described the performance in the video segment where poor leadership and team management caused operational breakdown:

Nurse 2: It’s noisy.

Nurse 3: We’re still not defibrillating. There’s still no clear direction—probably because there’s no team leader. Apart from compressions and airway, there’s not much else happening.

Nurse 2: Is it because the team leader is receiving a handover that’s inappropriate? She asked what was happening, and the first responder went around to give her the handover—so no one was giving them the direction. But they should be initiating without the team leader.

Nurse 3: Are they waiting for direction, or do they not actually know they need to shock early?

Nurse 2: I don’t know.

Nurse 3: That shouldn’t wait for a handover.

Nurse 3: I would say [they got] good knowledge of getting the pads on early, but no one knows the next step.

Nurse 2: Yep.

Nurse 3: And no one has claimed the defib role.

Nurse 2: Yep.

Nurse 3: And no one has claimed the defib role.

Nurse 2: Yep. I haven’t heard anyone claim roles.

Nurse 3: And I don’t know where the leader is. No one is ensuring roles are being done or covered.

(StandardCase2_Pair2)

**Fig. 7:**
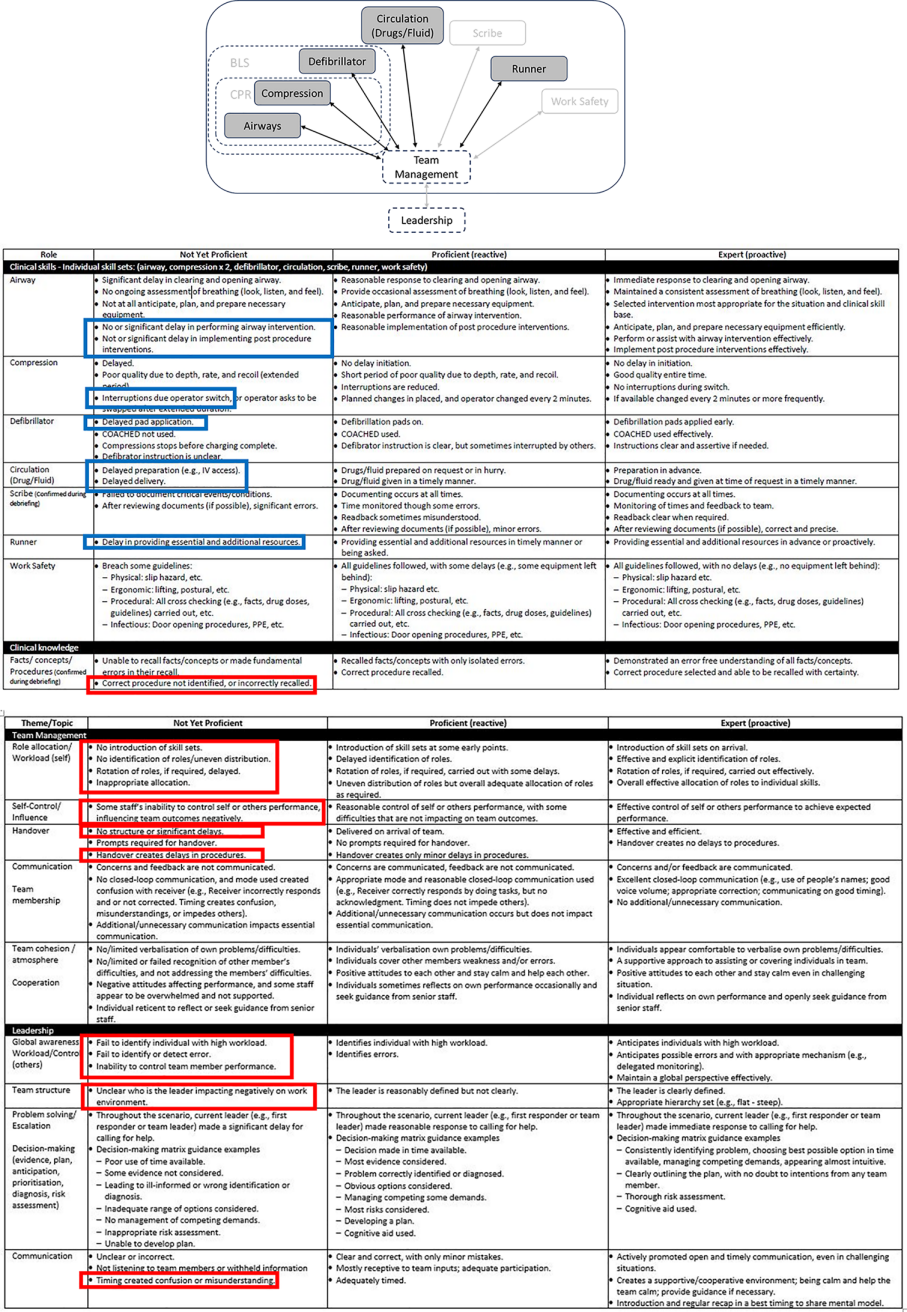
PRM-Resus example: The lack of clear leadership and failure to introduce team members’ skillsets at the beginning (red font) were identified as the root cause of the chaotic situation, resulting in poor role performance and delayed intervention (blue)

This discussion highlights how the absence of clear leadership—compounded by weak team coordination and knowledge gaps—can trigger cascading failures in performance. Figure 7 demonstrates how the PRM-Resus enabled the expert pair to trace the breakdown in leadership and role execution across the scenario.

## Discussion

### Overview

Effective performance in Advanced Life Support (ALS) requires more than technical proficiency; it relies on the integration and synergy of both technical and NTS at individual and team levels [5, 24]. In this study, we developed and evaluated the PRM-Resus—a reflective tool designed to be used alongside Video Set B (Phase 3) as *training videos* in ALS training. This tool supports ALS novice learners in reviewing video-recorded performance to deepen their understanding of the complex interplay between technical and NTS, individual roles and team management, or team management and leadership. When combined with the ALS Team Model and training videos, the PRM-Resus helps learners and educators understand these performance elements’ interdependent and inseparable nature.

The PRM-Resus uses consistent language to describe behaviours across a three-level rating scale: not yet proficient, proficient and expert. These explicit behaviour descriptors (i.e., word pictures) offer detailed insights into how the execution of skills and behaviours are demonstrated at each level. This structured vocabulary fosters a shared understanding of performance standards, learning objectives, and observable actions across all competency levels [2, 11]. When used to reflect on training videos, the PRM-Resus allows learners and faculty educators to interpret performance using descriptive, specific criteria. This process supports the development of pattern recognition, error identification, and a clear understanding of effective strategies [2].

### Practical implications: improve SBT and faculty development

While existing tools for assessing ALS and resuscitation performance often focus on either technical skills or NTS, few provide a comprehensive view that spans both technical and NTS in individual and team contexts across diverse clinical scenarios. As noted by Peltonen et al. (2017), many existing tools failed to address the integrated nature of ALS performance domains. In contrast, the PRM-Resus—used with the ALS Team Model and training videos—guides learners through a structured evaluation process. It begins with individual task performance and extends to dynamic, team-based behaviours and their relationships. Team structure and organisation are critical success factors in resuscitation. When these are lacking, delays, errors, and omissions are more likely [2, 6, 10, 24].

Given that ALS and resuscitation teams are frequently assembled ad hoc, training must prepare participants to adapt flexibly and function effectively in dynamic team environments. Models such as the “pit crew” approach, which assigns predefined roles and tasks to team members, have been shown to enhance performance and efficiency in both in-hospital and out-of-hospital cardiac arrest scenarios [25–27]. However, Peltonen et al. [27] suggest that while such strategies can improve short-term outcomes, they may also foster task fixation, potentially limiting the flexibility required in unpredictable, high-pressure environments.

By clearly defining individual roles and outlining performance expectations, the ALS Team Model and PRM-Resus combined use helps learners understand their responsibilities in contexts. This improves role clarity, supports effective team assembly, and enhances overall team function. It also demystifies the demands of each role, equipping learners to function more adaptively in real-world resuscitation settings.

The PRM-Resus may also benefit simulation observers. Used during guided video review, the tool supports vicarious learning—an approach rooted in Bandura’s Social Learning Theory [28]. Operational learning has yielded outcomes comparable to hands-on participation [4, 29]; however, it remains underutilised in healthcare simulation. The PRM-Resus—alongside the ALS Team Model and training videos—offers a structure for observation and encourages active reflection during debriefing, especially for ALS novice learners. Additionally, the PRM-Resus could serve as a faculty development resource. Its structured vocabulary and clearly defined performance indicators may help onboard instructors and standardise performance assessments across faculty, ensuring moderation and consistency in training delivery.

### Methodological implications: cross-sector study

A key strength of this study lies in the interdisciplinary development of PRM-Resus and the ALS Team Model. Cross-sector collaboration between healthcare, aviation, and education professionals created unambiguous descriptions of performance standards and behaviours. The need for clarity—especially from aviation and education contributors unfamiliar with clinical contexts—promoted productive dialogues and refinements. The robust word pictures provide ALS novice learners with a concrete understanding of performance expectations [14]. This innovative approach, grounded in educational theory [17] and informed by training and assessment practices from multiple domains [12, 16], yielded a novel and comprehensive learning tool for ALS performance. How this tool is best utilised within the simulation training environment is yet to be determined and will require ongoing evaluation.

### Limitations

One limitation of PRM-Resus is that its comprehensiveness may limit its usability in real-time simulation, where rapid assessment is required. However, it may be more effective in post-simulation video review, where learners can pause, reply, and critically analyse performance [4, 30]. This approach provides learners with a structured opportunity to understand how role clarity, leadership, and team management influence overall team effectiveness—potentially priming them to learn more and reflect more meaningfully on their own and their teams’ performance during SBT.

Familiarisation with the tool’s structure and vocabulary may mitigate these limitations, especially when used in faculty training. For example, instructors could be introduced to the PRM-Resus as part of a simulation program onboarding, supporting a shared performance language and deeper understanding of technical and NTS integration. Additionally, the tool may be used as a moderation instrument to ensure consistency in feedback and assessment standards across programs.

This study itself has additional limitations. First, the study was conducted at a single site, which may limit generalisability. Second, the professional backgrounds of the healthcare researchers—who may hold preconceived notions of effective performance—could have influenced data interpretation. However, contributions from aviation and education collaborators likely offset this effect and added valuable objectivity. Finally, transferring aviation-based models to healthcare can be challenging and limited due to contextual differences [21, 31]. Although the PRM-Resus was developed through a rigorous, multilayered reflection process, it requires further validation in diverse clinical and training settings.

### Future directions

The PRM-Resus and the associated training video are currently implemented in a hospital simulation training facility. Early data suggest that these resources enhance learner development and strengthen instructor capabilities. The PRM-Resus may help modulate learner performance, support structured reflective debriefings, and inform the design and delivery of simulation activities. These effects are achieved by clarifying how technical skills and NTS interplay at the individual and team levels.

Future research will explore PRM-Resus’ applicability in other healthcare contexts beyond the original study site. It will also investigate how PRM-Resus can improve the facilitation of team-based learning. Although team dynamics were frequently noted as challenging, they require deeper investigation. This need was apparent when researchers or think-aloud participants focused primarily on individual roles during video reviews (Phases 1 and 4). Greater emphasis on team and micro-team analysis is needed in both training and assessment. The emerging role of micro-teams in resuscitation—such as airway–compression–defibrillation sub-units—presents a valuable area for future exploration.

## Conclusion

The PRM-Resus is a novel, structured tool developed to provide a clear framework for assessing and enhancing understanding of performance in ALS. Using the ALS Team Model and training videos guides users to systematically consider the clinical skills of individual roles, clinical knowledge, team management, and leadership factors that collectively influence team performance in high-stakes scenarios.

While the tool shows strong potential to support learning and reflection in SBT, further evaluation is needed to determine how it can be most effectively implemented in diverse training environments. Future studies should investigate its usability, adaptability, and impact on both learner development and faculty facilitation within resuscitation education.

## Electronic supplementary material

Below is the link to the electronic supplementary material.

Supplementary Material 1

Supplementary Material 2

## Data Availability

The data that support the findings of this study are available from the corresponding author but restrictions apply to the availability of these data, which were used under license for the current study, and so are not publicly available. Data are, however, available from the authors upon reasonable request.

## Footnotes

1 Authors acknowledge that there are various terms describing these skills in the healthcare literature. NTS is purposely used in this paper as it compliments to the wider context including aviation.
