# Supplementary material for: Developing a learning tool for advanced life support and resuscitation: Performance Reflection Model for Resuscitation (PRM-Resus)
Source: BMC Med Educ. 2025 Jul 4;25:1001. doi: 10.1186/s12909-025-07509-9 (PMC12231619; doi:10.1186/s12909-025-07509-9)

# ***Appendix A. Think-aloud study setting***

Think-aloud study set up. Two participants watch the ALS video. Camera 1 captures the results recorded in the work area while synced Camera 2 captures participants and exact video segment being discussed on laptop.


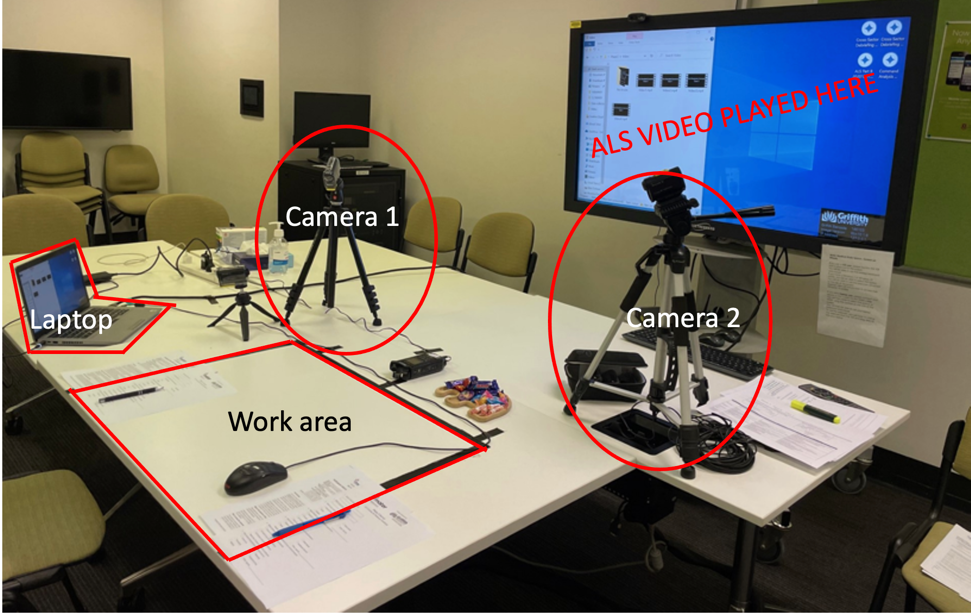

Supplement: Supplementary file 1 — Supplementary Material 1 [file 12909_2025_7509_MOESM1_ESM.docx]
