# Supplementary material for: Developing a learning tool for advanced life support and resuscitation: Performance Reflection Model for Resuscitation (PRM-Resus)
Source: BMC Med Educ. 2025 Jul 4;25:1001. doi: 10.1186/s12909-025-07509-9 (PMC12231619; doi:10.1186/s12909-025-07509-9)
Supplement: Supplementary file 2 — Supplementary Material 2 [file 12909_2025_7509_MOESM2_ESM.docx]

**Appendix B. Supplemental Quote Examples for Findings**

Quote 1:

*Nurse3: You know what I'd like to see is that the airway and the compressions.*

*Nurse2: Become a micro team.*

*Nurse3: ...the compression person, yeah, so they're each checking each other. So, compression is saying if there's equal chest rise and fall, and the airway person is letting the person doing chest compressions know that they're effective.*

*Nurse2: Yeah, completely agree. I think that's a really good indicator of excellent performance when they are providing feedback to each other. They always become a micro team.*

(StandardCase1_Pair 2: Original video sample: 1:14-2:04)

Quote 2:

*Nurse3: The person doing compression was up on the bed anyway.*

*Nurse2: And No.7 is very tall.*

*Nurse3: Yes. And if No.7 was going to intubate, the bed would have to be up anyway.*

*Nurse2: Yeah. So, I feel like it's a safety...*

*Nurse3: Yeah, because also even if they're standing on the step, it's quite low, so the person doing compressions is actually over leaning.*

*Nurse2: Yeah, over leaning, which means that they're not getting adequate recoil. So, I think it's safety and I'm going to put it in here. And I think it fits with PPE as an... I mean, PPE is very specific for the pandemic sessions, but as an overall safety, PPE would be...*

*Nurse3: Well, could that be a safety... yeah.*

(Standard Case 1_Pair 2: Overall comments)

Quote 3:

*… here (pointing at the screen), you know they've taken a team member essentially out of circulation by preparing for intubation. And even you know, having a medical officer hanging fluids, that person, that setting for intubation probably better place to be doing the fluid and drug management. So, I'm yeah, just curious as to how they've decided that that was the best use of their workforce.*

(Phase1_Co-Analysis1: 32:45-33:07)
